# Research on Aviation Safety Prediction Based on Variable Selection and LSTM

**DOI:** 10.3390/s23010041

**Published:** 2022-12-21

**Authors:** Hang Zeng, Jiansheng Guo, Hongmei Zhang, Bo Ren, Jiangnan Wu

**Affiliations:** 1 Equipment Management & UAV Engineering College, Air Force Engineering University, Xi’an 710051, China; 2 Science and Technology on Electro-Optic Control Laboratory, Luoyang 314000, China

**Keywords:** aviation safety, inducement of accident, prediction, adaptive sparse group lasso, long short-term memory

## Abstract

Accurate prediction of aviation safety levels is significant for the efficient early warning and prevention of incidents. However, the causal mechanism and temporal character of aviation accidents are complex and not fully understood, which increases the operation cost of accurate aviation safety prediction. This paper adopts an innovative statistical method involving a least absolute shrinkage and selection operator (LASSO) and long short-term memory (LSTM). We compiled and calculated 138 monthly aviation insecure events collected from the Aviation Safety Reporting System (ASRS) and took minor accidents as the predictor. Firstly, this paper introduced the group variables and the weight matrix into LASSO to realize the adaptive variable selection. Furthermore, it took the selected variable into multistep stacked LSTM (MSSLSTM) to predict the monthly accidents in 2020. Finally, the proposed method was compared with multiple existing variable selection and prediction methods. The results demonstrate that the RMSE (root mean square error) of the MSSLSTM is reduced by 41.98%, compared with the original model; on the other hand, the key variable selected by the adaptive spare group lasso (ADSGL) can reduce the elapsed time by 42.67% (13 s). This shows that aviation safety prediction based on ADSGL and MSSLSTM can improve the prediction efficiency of the model while keeping excellent generalization ability and robustness.

## 1. Introduction

The aviation industry is of great economic value, and to adapt to the current demand for intelligent and refined safety management, the aviation safety mitigation strategy has been shifting from a reactive to a proactive and predictive method. Therefore, it is greatly significant to clarify the causal mechanisms of aviation accidents and make corresponding early warning measures to promote the development of aviation safety.

Accurate aviation safety prediction is the basis of effective accident early warning, which has become a hot topic of research today. Recently, the aviation safety prediction method used specific machine learning methods to train the features of historical causes and accident samples, construct a mathematical analytical model, and measure the change trend of the safety level. For example, Liang et al. [1] used the BP (backpropagation) neural network model to predict the monthly incidents per 10,000 flight hours of an airline. Puranik et al. [2] built an online predictive model based on a Random Forest regression algorithm to predict landing performance metrics. Rosa et al. [3] constructed an aviation safety risk assessment model based on the Bayesian inference mechanism. Lukacova et al. [4] proposed a model for accident severity prediction based on classification and regression trees. Zhang et al. [5] proposed a combined method involving SVM (support vector machine) and deep neural networks to qualify the risk. Qiao et al. [6] constructed the RBF (radial basis function) neural network model to predict the hard landing. Machine learning not only has strong self-learning ability and robustness but also can better fit the nonlinear relationships among complex variables. However, the input variables of traditional machine learning models are generally regarded stand-alone, that is, the temporal features are not fully taken into consideration, which adds interference to the prediction results. To fully extract the temporal features, Xiong et al. [7] used LSTM neural network model to train and predict the sign data of American bird strike accidents. However, single-layer structures are adopted in that paper, and it’s difficult to accurately integrate the nonlinear relationships. Zhou et al. [8] used the ACARS (aircraft communications addressing and reporting system) accident report record as the research object, using the LSTM model to effectively capture the long-term dependency of samples and enhance the accuracy and robustness of safety measurement. Zhang [9] applied sequential deep learning techniques based on LSTM to perform a prognosis of adverse events. While the aviation accident data is typically a small sample, it’s difficult to efficiently learn sample characteristics by peer-to-peer prediction.

In addition to the selection of prediction methods, the selection of input variables affects the predicted effect. The classic accident inducement identification model is represented by the SHEL [10] model, REASON [11], HFACS [12] (human factors analysis classification system), OHFAM [13] (occurrence human factors analysis model) and 24 model [14]. The expansion of the inducement index is theoretically conducive to obtaining a more accurate causality description but brings the curse of dimensionality accordingly: the higher the dimensions of the input set are, the efficiency of safety prediction will be greatly reduced. Existing studies have led to some explorations in variable selection: Paulet al. [15], in the form of a questionnaire interview, intuitively modeled the greatest threat from the perspective of pilots. Cui et al. [16] used DEA (data envelope analysis) and a Malmquist index to calculate the civil aviation safety efficiency of Chinese airlines from 2008 to 2012, it proved that the quality of personnel training is the most important factor affecting aviation safety. In view of the high-dimensional samples with multiple collinearities, some scholars have tried to introduce the LASSO penalty term in the classic regression model. LASSO can greatly compress the value of independent variables to dilute the high-dimensional aviation data, which verifies the feasibility in the field of fuel consumption [17]. However, there is still a blank in the inducement of aviation accident variable selection. Meanwhile, the classic LASSO punishes the coefficient of key variables consistently, which weakens the feature identification of those key variables.

In view of the status that existing methods have an insufficient description of the importance of aviation accidents and low prediction efficiency, this paper proposes a new method of aviation safety prediction based on variable selection and LSTM. The contributions of the proposed method can be described as follows: (a) the dimensions of the security sample data are sufficiently reduced [18] by ADSGL, which reduces the cost of a running predictor and the elapsed time. (b) the multistep stacked LSTM model is constructed to explore the deep temporal features and complex nonlinear relationship of aviation safety samples and achieve smaller RMSE compared with the original model.

The rest of this paper is organized as follows: Section 2 describes the required individual algorithms and the process for building the proposed methods. Section 3 demonstrates a case study, followed by the training process for the constructed model. Section 4 provides a comparative analysis to verify the feasibility and effectiveness of the proposed method, and Section 5 presents the conclusions.

## 2. Proposed Aviation Safety Prediction Method

### 2.1. The Whole Process of the Proposed Method

**Figure 1:**
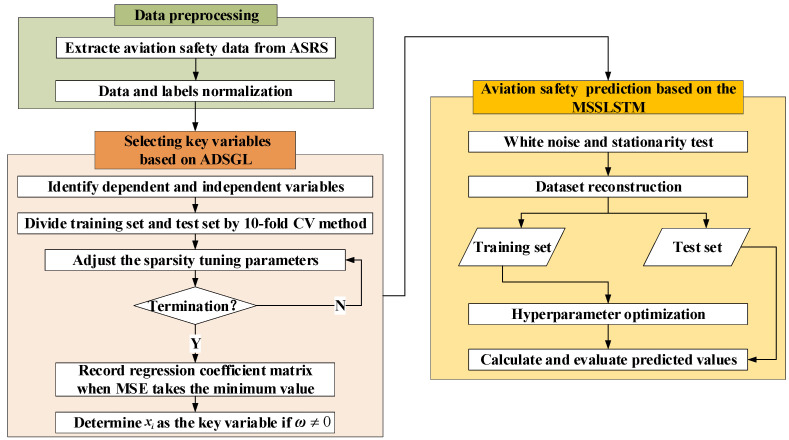
Flow chat of the ADSGL-MSSLSTM method.

The process of the ADSGL-MSSLSTM method is depicted in Figure 1. The detailed descriptions are given as follows:

(1) Data preprocess. Firstly, the aviation safety data are collected by the type of incidents from ASRS [19] reporting records, thus the text data is converted into structured data. Next, the aviation safety data is normalized into the range of [0, 1] to eliminate the interference of dimensional differences in data analysis.

(2) Key variables selection based on ADSGL. In view of the fact that there are too many manipulated variables to efficiently build predictive models, ADSGL is used to select key variables. The selected key variables are regarded to have the most interpretable information for incidents, which effectively reduces the operating cost and improves the calculation efficiency.

(3) Aviation safety prediction based on the MSSLSTM. In view of the fact that the predictors ignore the temporal dependence, which reduces the interpretability and accuracy, a multistep prediction model of aviation safety based on a multi-layer LSTM is constructed. Firstly, the white noise and stationarity of the data are tested. Secondly, the sample set is reconstructed to match the supervised learning mode. Thirdly, to enhance the ability of nonlinear fitting and temporal characteristic extraction, the hidden layer depth and learning step are adjusted, as well the learning error minimization is the criterion of optimal parameter establishment.

### 2.2. Selection of Aviation Safety Inducement Variables Based on ADSGL

#### 2.2.1. LASSO Penalty Operator

For the classic linear regression model [20] for aviation safety level, we defined it by

(1) $$y=X\omega +\varepsilon =\sum_{i=1}^{m}X_{i}\omega^{\left(i\right)}+\varepsilon$$

where $y={\left(y_{1},y_{2},\ldots ,y_{n}\right)}^{T}$ is the *n*-dimensional response variable vector, and $X=(X_{1},X_{2},\ldots ,X_{l})$ is the n*l-dimensional explanatory variable matrix. $\omega ={\left((\omega^{\left(1\right)}),(\omega^{\left(2\right)}),..,(\omega^{\left(l\right)})\right)}^{T}$ is the regression coefficient matrix, the distribution of error is followed as $\varepsilon \sim N(0,\sigma^{2}I_{n})$.

The sum of the error is taken as the loss function, the penalized likelihood estimation [21] (objective function) is defined as

(2) $$\hat{\omega }=\underset{\omega }{\mathrm{argmin}}\{\frac{1}{2}{\left\|y-\sum_{i=1}^{m}X_{i}\omega^{\left(i\right)}\right\|}_{2}^{2}+\sum_{i=1}^{m}P_{\lambda }(\left|\omega^{\left(i\right)}\right|)\}$$

where ${\left\|\cdot \right\|}_{2}^{}$ is the 2-Norm, penalized expressions for a single input variable are usually expressed as $P_{\lambda }(\left|\omega \right|)=\lambda {\sum_{i=1}^{l}\left|\omega^{\left(i\right)}\right|}^{q}$, 0 < *q* ≤ 2. The penalty term is called the LASSO penalty when *q* = 1. *λ* is the adjustable coefficient, which can be adjusted to obtain the optimal solution and scale the penalty term appropriately.

The advantages brought by the LASSO penalty into the inducements variable selection of aviation incidents can be illustrated as follows [22].

(1) Increase the sparsity of the regression coefficient matrix. Sparsity refers to the existence of several sample points with a value of 0, which conforms to the actual selection of incident inducement variables; when a candidate variable is an irrelevant interference variable, the corresponding regression coefficient is 0. The $\omega^{\left(i\right)}$ is strictly equal to 0 when λ takes a large value, so as to realize the sparsity of the coefficient matrix and to achieve the variable selection.

(2) Increase in model stability. The input variable matrix is often followed by multicollinearity, and the obtained result may be non-inferior, which only satisfies the local optima. Daubechies, Defrise and De Mol [23] prove that any penalty for the coefficient matrix satisfying 1 ≤ *p* ≤ 2 can make the solution of the original model more stable.

However, the classic LASSO algorithm still has obvious defects: when making a univariate selection, LASSO agrees with the shrinkage multiple of the regression coefficient, which will impose more penalty on the coefficient term, resulting in the inconsistency and bias of the dimensionality reduction model.

#### 2.2.2. Construction of the Algorithm for Aviation Safety Variable Selection

To address some limitations of the classic LASSO algorithm, this paper adopts a variable selection method based on the adaptive sparse group LASSO: when optimizing the minimum loss function, the function is univariate, that is, to adapt to the various inducement variables and the vastly different punishment strategy features, only one variable is optimized each time and cycled repeatedly until all variables converge.

*l* explanatory variables are divided into *N* non-overlapping groups. The objective function of ADSGL is formulated as

(3) $${\hat{\omega }}^{\mathrm{ADSGL}}=\mathrm{argmin}\frac{1}{2}{\left\|y-\sum_{i=1}^{N}X_{i}\omega^{\left(i\right)}\right\|}_{2}^{2}+\lambda (1-\alpha )\sum_{i=1}^{N}\tau_{i}({\left\|\omega^{\left(i\right)}\right\|}_{2})+\lambda \alpha \sum_{i=1}^{N}{\left(\xi^{\left(i\right)}\right)}^{T}\left|\omega^{\left(i\right)}\right|$$

where ${\left[\tau \right]}_{1\times N}$ is the weight matrix explaining the group variables, ${\left[\xi \right]}_{1\times l}$ is the weight matrix explaining the univariate. The weight matrix introduced by ADSGL can effectively reduce the penalty of the biggish coefficient, which keeps the model consistent and unbiased and improves the accuracy of variable selection. The algorithmic steps are summarized as follows:

Step 1 the initial value of the regression coefficient is set as

(4) $$\hat{\omega }=({\left(\omega_{0}^{\left(1\right)}\right)}^{T},{\left(\omega_{0}^{\left(2\right)}\right)}^{T},\ldots ,{\left(\omega_{0}^{\left(N\right)}\right)}^{T})$$

Step 2 for *k* = 1, 2, …, *N*, if *k* satisfies the (5), then ${\hat{\omega }}^{\left(k\right)}=0$; Otherwise, turn to the Step 3.

(5) $$\;\left\|K(X_{k}^{T}(y-\sum_{i\neq k}X_{i}{\hat{\omega }}^{{}_{\left(i\right)}}),\alpha \lambda \xi^{\left(k\right)})\right\|\leq (1-\alpha )\lambda \tau_{k}$$

K(⋅) is defined as

(6) $$K(a,b)=(K{\left(a,b\right)}_{1},K{\left(a,b\right)}_{2},\ldots ,K{\left(a,b\right)}_{m})$$

(7) $$K{\left(a,b\right)}_{i}=sign(a_{i})\{\max\{0,\left|a_{i}\right|-b_{i}\}\}$$

Step 3 In the *k* th group of the regression coefficient, for $j=1,2,\ldots ,l_{k}$, if *k* satisfies the (8), then ${\hat{\omega }}_{j}^{\left(k\right)}=0$; Otherwise, turn to the Step 4.

(8) $${\left(X_{ki}\right)}^{T}[(y-\sum_{i\neq k}X_{i}{\hat{\omega }}^{{}_{\left(i\right)}})-\sum_{p\neq j}{\left(X_{kp}\right)}^{T}{\hat{\omega }}_{p}^{\left(k\right)}]\leq \alpha \lambda \xi_{j}^{\left(k\right)}$$

Step 4 ${\hat{\omega }}_{i}^{\left(k\right)}$ is taken in (9) to solve the optimized regression coefficient values.

(9) $${\overset{\;}{\omega }}_{j}^{\left(k\right)}=\underset{\omega_{j}^{\left(k\right)}}{\mathrm{argmin}}\{\frac{1}{2}{\left\|y-\sum_{i=1}^{N}X_{i}\omega^{\left(i\right)}\right\|}_{2}^{2}+(1-\alpha )\lambda \tau_{{}^{k}}{\left\|\omega^{\left(k\right)}\right\|}_{2}+\alpha \lambda \xi^{\left(k\right)}\left|\omega^{\left(k\right)}\right|\}$$

Step 5 The threshold of absolute error is set and Step 2~Step 4 is cycled until convergence. The convergence algorithm is expressed as

(10) $$f(\omega )=f_{0}(\omega )+\sum_{i=1}^{N}f_{i}(\omega )$$

(11) $$f_{0}(\omega )=\frac{1}{2}{\left\|(y-\sum_{i=1}^{N}X_{i}\omega^{{}_{\left(i\right)}})\right\|}_{2}^{2}\;$$

(12) $$f_{i}(\omega )=(1-\alpha )\lambda \tau_{i}({\left\|\omega^{\left(i\right)}\right\|}_{2})+\alpha \lambda {\left(\xi^{\left(i\right)}\right)}^{T}\left|\omega^{\left(i\right)}\right|$$

where *f*_0_ is a strictly convex function and $f_{j}(\omega )$ can be differentially. The algorithm avoids premature convergence and falling into a local optimum [24], and dynamically adjusts the penalty term according to the size of the regression coefficient, effectively reducing the calculation errors.

### 2.3. Aviation Safety Prediction Based on LSTM Neural Network

#### 2.3.1. LSTM Architecture

**Figure 2:**
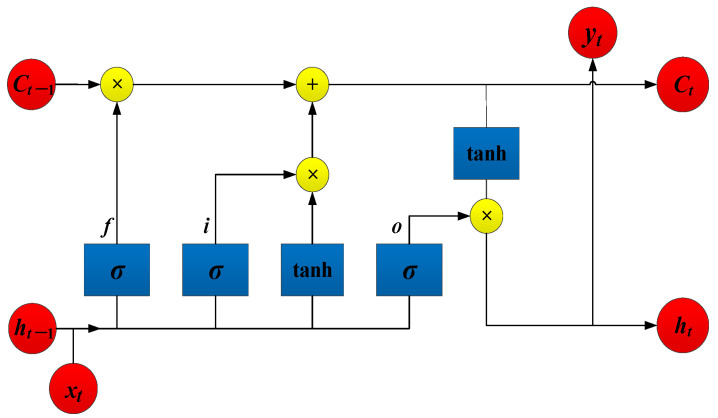
The network architecture of LSTM.

To evaluate the selected key variables in improving the prediction accuracy, the LSTM architecture is used as an aviation safety predictor. The LSTM architecture has a memory unit and forget gate, which can update the cell state in real-time: the previous output (*h_t_*_−1_) and the current input (*x_t_*) enter the forget gate and determines how much information is forgotten from the previous state (*c_t_*_−1_). Accordingly, the input gate determines how much the updated state is retained in the current state (*c_t_*); the output gate determines how much information the ***c_t_*** export, thus continuously updating the state parameters over time. The network architecture of LSTM is shown in Figure 2.

Where *f* is the forget gate, *i* is the input gate, and *o* is the output gate. σ is the incentive function, which generally takes the sigmoid function. The tanh is a hyperbolic tangent function. sigmoid and hyperbolic tangent function can be implemented as

(13) $$\sigma (x)=\frac{1}{1+e^{x}}$$

(14) $$\tanh(x)=\frac{e^{x}-e^{x}}{e^{x}+e^{x}}$$

The updated formulas [25] for each gate and cell state can be implemented as

(15) $$f_{t}=\sigma [W_{{}_{f}}^{t},U_{{}_{f}}^{t}]\cdot {\left[X_{t},h_{t-1}\right]}^{T}+b_{{}_{f}}^{t})$$

(16) $$i_{t}=\sigma ([W_{i}^{t},U_{{}_{i}}^{t}]\cdot {\left[X_{t},h_{t-1}\right]}^{T}+b_{i}^{t})$$

(17) $$o_{t}=\sigma ([W_{{}_{o}}^{t},U_{{}_{o}}^{t}]\cdot {\left[X_{t},h_{t-1}\right]}^{T}+b_{{}_{o}}^{t})$$

(18) $${\tilde{c}}_{t}=\tanh([W_{{}_{c}}^{t},U_{{}_{c}}^{t}]\cdot {\left[X_{t},h_{t-1}\right]}^{T}+b_{{}_{c}}^{t})$$

(19) $$c_{t}=i_{t}\cdot {\tilde{c}}_{t}+f_{t}\cdot c_{t-1}$$

(20) $$h_{t}=o_{t}\cdot \tanh(c_{t})$$

where *W* is the weight of *x_t_*, *U* is the weight of *h_t_*_−1_, *b* is the biases, ${\tilde{c}}_{t}$ is the critical state of the cell.

When processing the RNN model, the iterations of BPTT (backpropagation through time) increase due to the higher amounts of steps, which is most likely to initiate the gradient disappearance so that each parameter cannot be accurately updated. However, the unique gate structure of LSTMs can better alleviate such problems. BPTT can be implemented as

(21) $$\frac{\partial J_{t}}{\partial W}=\sum_{K=0}^{t}\frac{\partial J_{t}}{\partial h_{t}}\cdot \frac{\partial h_{t}}{\partial c_{t}}\cdot \frac{\partial c_{t}}{\partial c_{t-1}}\cdots \frac{\partial c_{2}}{\partial c_{1}}\cdot \frac{\partial c_{1}}{\partial W}=\sum_{k=0}^{t}\frac{\partial J_{t}}{\partial h_{t}}\cdot \frac{\partial h_{t}}{\partial c_{t}}\cdot \prod_{h=k+1}^{t}\left(\frac{\partial h}{\partial c_{h-1}}\right)\cdot \frac{\partial c_{k}}{\partial W}$$

where *J_t_* represents the cumulative training loss from the start to the current moment. In RNN: $\frac{\partial c_{h}}{\partial c_{h-1}}\approx \sigma \prime (W-X+b)=\sigma (1-\sigma )$ and the value is [0, 1/4]. While in LSTM: $\frac{\partial c_{h}}{\partial c_{h-1}}\approx \sigma$ and the value is [0, 1]. When the training time is too long, the cumulative effect makes the training gradient of RNN tend to 0, greatly reducing the learning rate. LSTM can control $\prod_{h=k+1}^{t}\frac{\partial c_{h}}{\partial c_{h-1}}\approx \sigma$ at about 1, to prevent the gradient disappearance or explosion, and ensure the accurate update of parameters.

#### 2.3.2. Prediction Process

(1) Stationarity and white noise test

The research object of the LSTM predictor is a temporal aviation safety sample, so the stationarity and white noise of the sample should be tested first, and the tested sample can be analyzed and predicted by the predictor.

**Figure 3:**
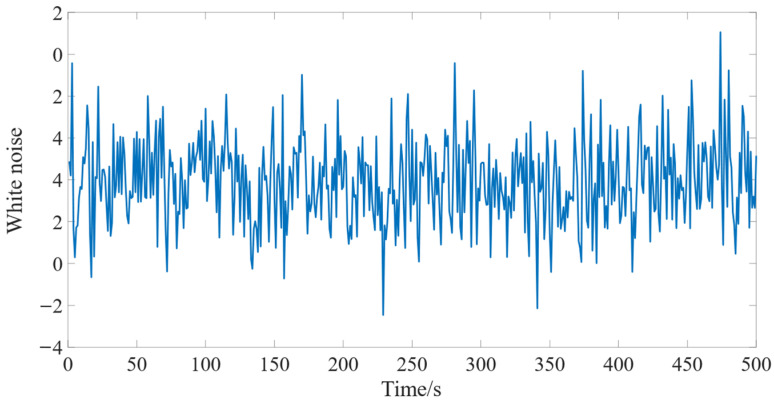
Sequence diagram for white noise.

A stationary sequence refers to a temporal sample with no obvious volatility, trend, or cyclicity. A white noise sequence is randomly generated and mutually uncorrelated, and it is characterized by 3 features:

1) *E*(*x_t_* = 0); 2) *D*(*x_t_*) = *σ*^2^; 3) Cov(*x_t_*, *x_t + k_*) = 0 (*k* ≠ 0). A schematic drawing of white noise sequence is shown in Figure 3.

(2) Refactoring the data

Input and output feature vectors (*x_t_*, *y_t_*) are refactored and fit into the sequence-to-sequence learning in the LSTM network: *x_t_* and *y_t−_*_1_ (*t* = 2,…, *n*) are merged as.

(22) $$\begin{array}{l} X\prime =\{(x_{2},y_{1}),(x_{3},y_{2}),\ldots ,(x_{n},y_{n-1})\}\\ Y^{\prime }=\{y_{2},y_{3},\ldots ,y_{n}\} \end{array}$$

The training set is also divided according to each step size.

(23) $$\left\{(X_{t-p}^{\prime },\ldots ,X_{t-1}^{\prime }),(Y_{t}^{\prime },\ldots ,Y_{t+q}^{\prime }),t=p+1,\ldots ,n-q\right\}$$

The test set is refactored as.

(24) $$\left\{(X_{n-p-q+1}^{\prime },\ldots ,X_{n-q}^{\prime }),(Y_{n-q+1}^{\prime },\ldots ,Y_{n-1}^{\prime })\right\}$$

where *n* is the sum of the time steps, *p* is the length of the input sample, and *q* is the length of the output sample.

(3) Constructing a multistep stacked predictor of aviation safety

**Figure 4:**
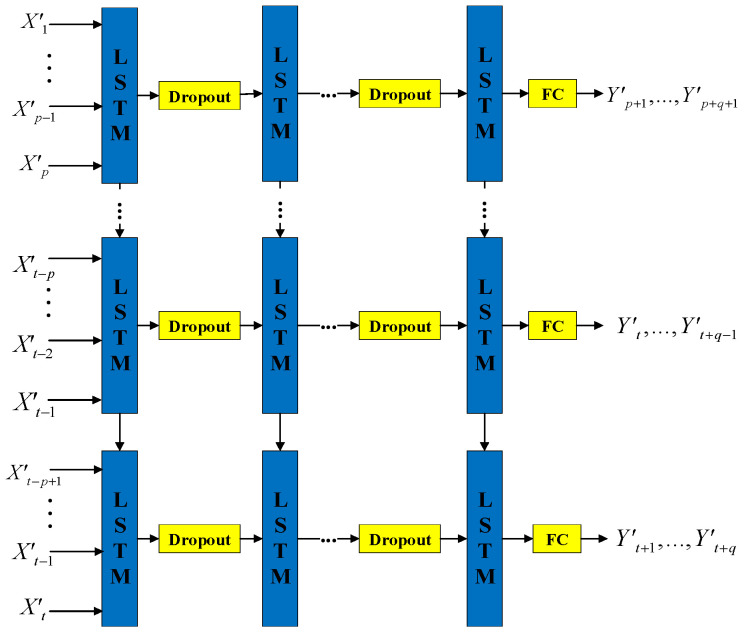
Aviation safety prediction model based on MSSLSTM.

Peer to peer prediction adopted by the original LSTM cannot accurately describe the changes of the aviation safety level. Additionally, a single layer network lacks the ability to capture the temporal dependence of dataset. To address the above problems, the training layers and step size of the classic LSTM model are adjusted. The MSSLSTM model is constructed in turn, as shown in Figure 4.

In the same training batch, the number of hidden layers neuron is set as *k*, and the number of hidden layers is set as *l*. The output of the previous hidden layer is used as the input of the current layer, the dropout layer is set between adjacent hidden layers to reduce the model complexity, and the last hidden layer and output termination are fully connected [26]. Finally, the *n*-dimensional output vector is activated by the softmax function. The fully connected layer can be implemented as.

(25) $${\left[{\overset{⌢}{y}}_{i}\right]}_{q\times 1}=\sum_{\begin{array}{l} j\in [1,q]\\ K\in [1,p] \end{array}}{\left[\omega_{jk}\right]}_{q\times p}\cdot {\left[{\overset{⌢}{h}}_{K}\right]}_{p\times 1}$$

The multistep stacked LSTM predictor not only increases the input sizes, which helps calculate the digital features of multiple historical samples in the input gate, improving the usage of existing samples but also increases the length of the output sample, which helps to describe the change trend of the aviation safety level more intuitively. Compared with the original LSTM model, the multistep stacked LSTM predictor strengthens the cell’s learning performance on the temporal features, explains the trendy changes in safety level more visually, and helps conduct better forward-looking and real-time prediction. Additionally, the stacked architecture not only greatly strengthens the deep learning ability, but improves the robustness and adaptability of the predictor.

(4) Optimizing hyperparameters

The learning rate is optimized by the Adam algorithm with weight decay, the number of hidden layers and nodes are ergodic by step search, the MAE (mean absolute error) of the training results are compared under different parameter combinations, and it is recorded when the MAE takes the minimum value.

MAE can be implemented as

(26) $$\mathrm{MAE}=\frac{\sum_{i=1}^{n}\left|y_{i}-y_{{}_{i}}^{\prime }\right|}{n}$$

where $y_{i}$ is the test value, $y_{{}_{i}}^{\prime }$ is the predicted value.

The Adam optimization process is as follows.

Firstly, the first-order and second-order moment estimation of the gradient is calculated:

(27) $$m_{t}\leftarrow \beta_{1}m_{t-1}+(1-\beta_{1})\cdot g_{t}$$

(28) $$v_{t}\leftarrow \beta_{2}v_{t-1}+(1-\beta_{2})\cdot g_{t}^{2}$$

where $\beta_{1}$ and $\beta_{2}$ are decay rates for the first-order and second-order moment estimates.

Secondly, the deviation term of the correction moment estimate is calculated:

(29) $${\overset{⌢}{m}}_{t}\leftarrow m_{t}/(1-\beta_{1}^{t})$$

(30) $${\overset{⌢}{v}}_{t}\leftarrow v_{t}/(1-\beta_{2}^{t})$$

Finally, the learning rate update value is calculated based on the correction:

(31) $$\Delta \theta =-\eta_{0}\frac{{\overset{⌢}{m}}_{t}}{\sqrt{{\overset{⌢}{v}}_{t}}+\delta }$$

where $\eta_{0}$ is the initial learning rate. Adam controls the updated learning rate in the measurable range, and accelerates the collection speed and ensures the robustness of the model.

(5) Model accuracy evaluation

To intuitively evaluate the accuracy of the prediction model, single layer-multistep LSTM (LSTM′), the original LSTM, ML-RNN, TCN (temporal convolutional network), DT (decision tree), SVM, and ARMA (autoregressive moving average) predictors are used as control models to compute in the same experimental environment. RMSE is used as the accuracy evaluation indicator. RMSE can be implemented as

(32) $$\mathrm{RMSE}=\sqrt{\frac{\sum_{i=1}^{N}{\left(y_{i}-y_{{}_{i}}^{\prime }\right)}^{2}}{n}}$$

## 3. Examples of Aviation Safety Predictions Based on Variable Selection and LSTM

### 3.1. Data Preprocessing

The study sample was taken from ASRS. The complete report record [27] includes Time, Environment, Aircraft, Component (faulty parts, accident recorded with mechanical failure), Person, Events, Assessments, and Synopsis. The text data example is shown in Table 1.

According to the description of Assessments and Synopsis in the reports, the variables of the accident dataset can be classified into 12 input variables: aircraft (A), company policy (CP), procedure (P), weather (W), communication breakdown (CB), confusion (C), distraction (D), human-machine interface (HMC), situational awareness (SA), time pressure (TP), training/qualification (TQ), workload (WL), and 1 output variable: minor accident (MA). Based on this, the monthly statistics of accidents from June 2010 to Nov 2020 were collected.

To eliminate the interference from dimensional differences [28], the dataset was normalized by min-max normalization. The normalized index values were scaled linearly between 0 and 1. The min-max normalization can be implemented as.

(33) $$x^{*}=\frac{x-x_{min}}{x_{max}-x_{min}}$$

where *x_max_* and *x_min_* represent the maximum and minimum values of the sample, *x* is the original value, x* is the normalized value. Normalized results are shown in Table 2.

The differences in value distributions were eliminated in the normalized sample, and the numerical features are retained by equal scale.

### 3.2. Selection of Key Variables Based on the ADSGL

#### 3.2.1. Setting of the Key Parameters of the Variable Selection Algorithm

The aircraft incident in Table 3 was selected as the dependent variable and denoted by $\{y_{t}\},t\in \left(0,138\right]$; The remaining 12 types of insecure events were selected as the independent variable and denoted by $\{x_{t}^{\left(i\right)}\},i\in (0,12],t\in \left(0,138\right]$. To improve the usage of the sample while avoiding overfitting [29], 10-fold cross-validation was used to divide the dataset. Given the actual sample size, the epoch was set at 100, and the number of groups was set at 2. To ensure the credibility of the experimental results, the variable selection was performed in the different relaxed variables (*α*). Parameters settings are shown in Table 3.

#### 3.2.2. Analysis of Experimental Results

Minimum MSE (mean square error) is selected as the final selection results. MSE can be implemented as.

(34) $$\mathrm{MSE}=\frac{\sum_{i=1}^{N}{\left(y_{i}-y_{{}_{i}}^{\prime }\right)}^{2}}{N}$$

**Figure 5:**
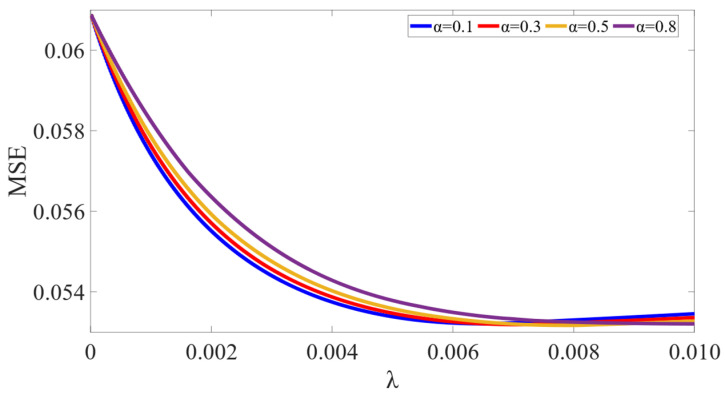
Changes of the validation MSE under the different relaxation variables.

The experimental results are shown in Figure 5.

**Figure 6:**
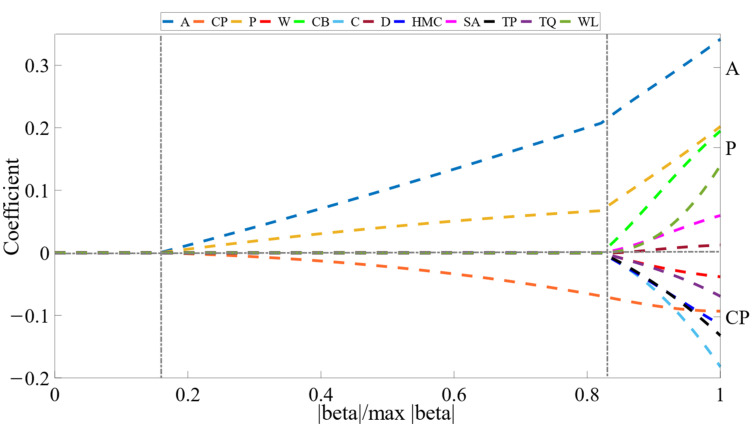
Lasso fitting coefficient tracer diagram as α takes 0.1.

Figure 5 shows the changes in the validation MSE with *λ* increasing. As α are set to 0.1, 0.3, 0.5, 0.8, the minimum MSE values in these 4 groups of experiments all are around 0.05, and the convergence speeds up as alpha increases, which reflects the good robustness of ADSGL in terms of error convergence, and a strong positive correlation is implicated between alpha and convergence speed. Lasso fitting coefficient tracer diagram as *α* takes 0.1 is shown in Figure 6.

From Figure 6, it can be seen that: One group of variables’ coefficients (W, CB, C, D, HMC, SA, TP, TQ, WL) converged at a higher rate compared with the other group (A, CP, P), which indicates that the variables in the latter group are still highly interpretable to the output variables at optimal penalty weight. The absolute values of coefficients, which in the first group converged, are depicted in Figure 6.

**Figure 7:**
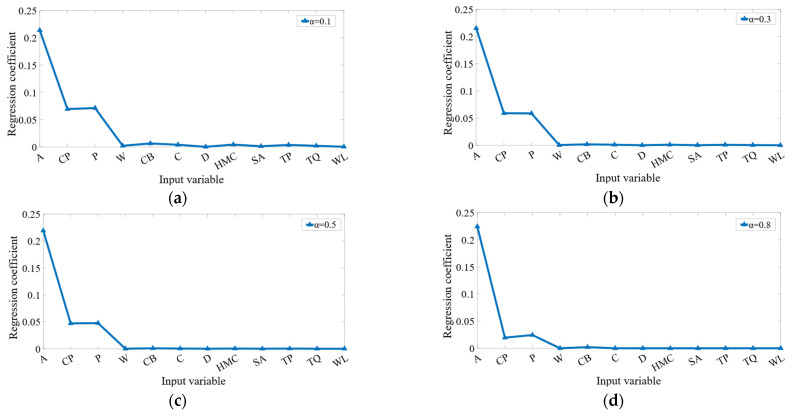
The absolute values of compressed regression coefficients as *α* set to (**a**) 0.1, (**b**) 0.3, (**c**) 0.5, (**d**) 0.8.

From Figure 7, it can be found that the values of compressed regression coefficients are relatively consistent when the α is adjusted, which demonstrates that the ADSGL method is robust in the contraction of regression coefficients. Furthermore, the common intersection set of the results of the four experimental are {A, CP, P}, the regression coefficients of which are non-zero in the largest penalty, having a greater impact on the MA. Therefore, those three variables are selected as the input variables of the aviation safety predictor.

### 3.3. Multistep Aviation Safety Prediction Based on the MSSLSTM Model

#### 3.3.1. Dataset Refactoring

After passing the white noise and stationarity test, the dataset was refracted and fit into the supervised learning form of the LSTM network: ***x_t_*** was merged with *y_t−_*_1_ (*t* = 2,…, *n*) into the feature set, which can be implemented as $X\prime =\{(x_{2},y_{1}),(x_{3},y_{2}),\ldots ,(x_{138},y_{137})\}$, and the output set was $Y^{\prime }=\{y_{2},y_{3},\ldots ,y_{138}\}$.

The training step was generally fixed in the learning process and set based on actual demand, given the significant temporal dependence of aviation accidents, the lengths of the input and output samples were set to 4: the historical data of the last 4 months were used to predict the safety level in the next 4 months. The training and test sets were divided accordingly:

$$\{(X_{t-4}^{\prime },\ldots ,X_{t-1}^{\prime }),(Y_{t}^{\prime },\ldots ,Y_{t+3}^{\prime }),t=5,\ldots ,131\},\{(X_{131}^{\prime },\ldots ,X_{134}^{\prime }),(Y_{135}^{\prime },\ldots ,Y_{138}^{\prime })\}$$

#### 3.3.2. Stability Test and White Noise

**Figure 8:**
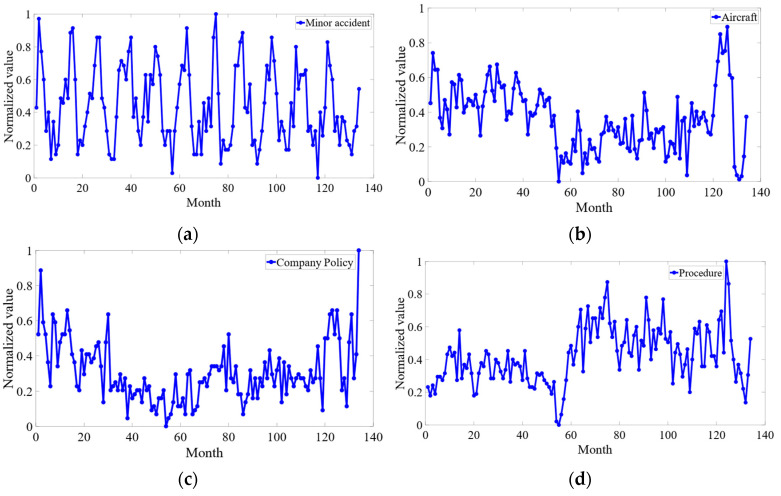
Sequence diagram for (**a**) MA, (**b**), A (**c**) CP, (**d**) P.

To visually present the trend features of the sample over time, each variable was analyzed. The sequence diagram for the insecure events is presented in Figure 8.

Figure 8a shows that the sample variance of the minor accident is stable overall. The sequence has little volatility, and no obvious trend and cyclicity, so it can be regarded as a stationary sequence. Additionally, the expectation for the sample is much larger than 0, which does not satisfy the autocorrelation property of a white-noise sequence. Thus, the sample passed the white noise test.

In turn, the other variables were observed in turn according to the sequence diagram, the results show that all input and output variables were stationary and non-white-noise sequences.

#### 3.3.3. Hyperparameter Optimization

To observe the predicted effect of the model at different batches, the number of nodes and number of layers were successively adjusted. To alleviate the overfitting, neurons were randomly discarded at 65% for each training session (dropout_rate = 0.65).

**Figure 9:**
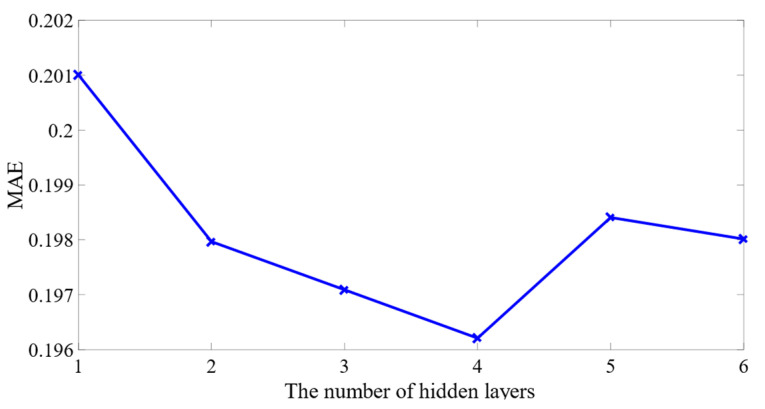
Changes of training accuracy under different number of layers.

Furthermore, we adjusted the number of layers, constructed the MSSLSTM aviation safety predictor, and traversed the training error (MAE). Figure 9 shows that the MAE decreases as the layer number increases to 4 and the minimum MAE is about 0.1962, which demonstrates that the stacked structure enhances the deep learning ability. While MAE increases as the layer number is over 4, the possible cause is overfitting due to model complexity. Thus, the number of hidden layers in this case was set as 4.

**Figure 10:**
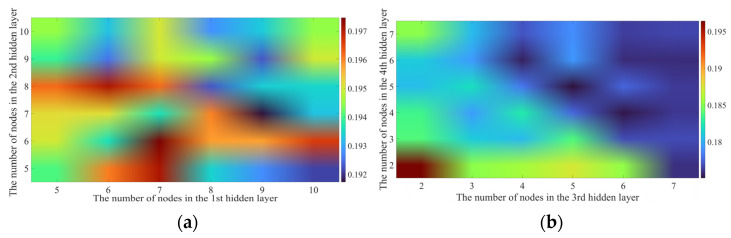
Changes of training accuracy under different number of nodes in the (**a**) 1st and 2nd hidden layer, (**b**) 3rd and 4th hidden layer.

Figure 10a shows the changes in training errors under the number of nodes combination of the first two layers. It shows that the global minimum MAE is about 0.1917, as the number of nodes were set as 9 and 7.

Based on the optimization of the nodes’ number in the first two layers, we adjusted the nodes’ number in the last two layers, training errors are accordingly shown in Figure 10b, the global minimum MAE is about 0.1751, as the number of nodes were set as 5, 5.

From the parameters described above, the optimal setting for the number of nodes was set as 9, 7, 5, 5.

## 4. Model Comparison

### 4.1. Effect of Predictors on the Experimental Results

**Figure 11:**
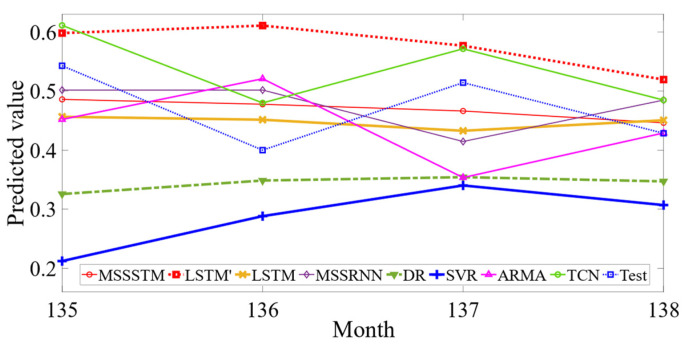
Comparison of the predictive model results.

The model proposed in this paper based on the MSSLSTM will be compared with prediction models such as LSTM′, LSTM, MSSRNN, TCN, DT, SVR, and ARMA. Those compared models were all used to predict the value of aviation safety level in the same experimental environment. The comparison experiment results of each model are shown in Figure 11.

From Figure 11, it can be seen that: (1) the MSSLSTM predictor outperforms the original LSTM in the fitting effect. (2) Recurrent neural networks (RNNs) are better than the ARMA model, as it demonstrates a better ability to recognise and understand trends by the memory units. (3) For DR and SVR, the prediction errors have a much wider range of deviations, compared with RNNs, which indirectly proved that RNNs are relatively robust. (4) TCN performs well to fit increasing and decreasing trends of predicted value but has a large relative error.

**Figure 12:**
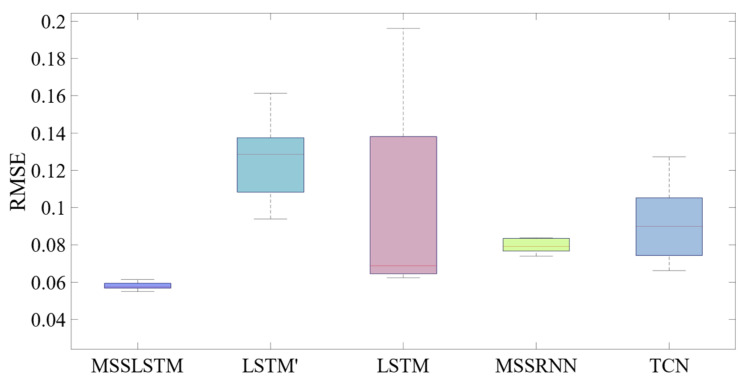
The RMSE distribution diagram of the ten-time experiments.

The RMSE distribution of the 10 experiments is recorded. As shown in Figure 12: (1) The error of MSSLSTM is the least, and RMSE is below 0.058, which is reduced by 41.977% and 28.37% compared with LSTM and TCN. This further proves that the robustness of the MSSLSTM proposed in this paper has been strengthened. (2) The accuracy of the MSSLSTM is slightly higher than that of the MSSRNN, which shows that gate structure helps improve the accuracy of multi-layers predictor.

### 4.2. Effect of Variable Selection Methods on the Experimental Results

To more intuitively verify the effectiveness and robustness of ADSGL for improving safety prediction efficiency, LASSO, Group LASSO, ridge regression (RR), and random forest (RF) are introduced into the proposed MSSLSTM predictor, respectively, and the input variables determined by the variable selection methods are tested. The RMSE, elapsed time (*t*), and the number of selected variables (Variable_number) are used as evaluation indicators.

The comparison experiment results of each model are shown in Table 4.

It can be seen from the comparison of Table 4 that: (1) The predicted RMSE of ADSGL is 0.058, which is significantly lower than that of the original operator (LASSO, RR), and the convergence error is the minimum. It shows that the accuracy of the proposed ADSGL is greatly improved, compared with the original operator. (2) The accuracy of the ADSGL is better than that of the RF, and the error decreased by more than 35.53%, which verifies the effectiveness of the penalty term in improving the accuracy. (3) Compared with the original LASSO, the elapsed time is shortened by nearly 13 s. It proves that the proposed ADSGL greatly improves elapsed efficiency. (4) The number of variables selected by ADSGL is 3, under the premise of ensuring prediction accuracy, the sample dimensions get fully reduced, by about 63.3%, with better interpretability.

## 5. Conclusions

This paper presents a new aviation safety prediction method based on ADSGL- MSSLSTM. In this study, ADSGL is used to select key variables based on coefficients’ shrinking at both the group level and univariate level, and penalize the coefficients differently depending on their adaptive weights. In this way, the evaluations are ensured to be unbiased and consistent, which improves selection model accuracy and interpretability. Subsequently, MSSLSTM is trained using a dataset after dimension reduction, and it’s then used to predict the changing trend in minor accidents. The multi-step stacked structure enhances the predictor by generalizing and analyzing complex temporal dependencies and nonlinear relationships, which helps further improve the prediction efficiency and robustness.

The feasibility of ADSGL-MSSLSTM is demonstrated by case studies of data collected in ASRS, from June 2010 to Nov 2020. Various existing methods are determined as control models. Experimental results indicate that the proposed method can efficiently conduct key variable selection and improve the prediction performance of aviation incidents.

As part of the future work, firstly, given the difference in safety among various models of aircraft, we will apply the proposed method to predict the safety level in a specific model, thus extracting more tailored safety information. Additionally, appropriately increasing the appropriate coefficient penalty will be adopted to speed up the error convergence rate while satisfying the accuracy demand.

## Figures and Tables

**Table 1:** An example of the safety report taken from ASRS.

Attribute	Content
Time/Day	Date: 202006 Local Time Of Day: 1201–1800
Place	Altitude. MSL.Single Value: 39,000
Environment	Flight Conditions: IMC Weather Elements/Visibility: Turbulence Weather Elements/Visibility: Rain
Aircraft	Make Model Name: Stratotanker 135 Crew Size. Number Of Crew: 2 Flight Plan: IFR Mission: Refueling Flight Phase: Cruise Route In Use: Direct
Component	None
Person	Location In Aircraft: Flight Deck Reporter Organization: Military Function. Flight Crew: Pilot Flying Function. Flight Crew: Captain Qualification. Flight Crew: Multiengine Qualification. Flight Crew: Instrument ASRS Report Number. Accession Number: 1745628 Human Factors: Workload
Events	Anomaly. Deviation-Altitude: Excursion From Assigned Altitude Anomaly. Deviation/Discrepancy-Procedural: Clearance Anomaly. Inflight Event/Encounter: Weather/Turbulence Anomaly. Inflight Event/Encounter: Loss Of Aircraft Control Detector. Person: Flight Crew When Detected: In-flight Result. Flight Crew: Took Evasive Action Result. Flight Crew: Requested ATC Assistance/Clarification Result. Flight Crew: Regained Aircraft Control Result. Flight Crew: Overcame Equipment Problem Result. Air Traffic Control: Provided Assistance
Assessments	Contributing Factors/Situations: Weather Primary Problem: Weather
Synopsis	KC-135 Captain reported experiencing temporary loss of control due to severe turbulence.

**Table 2:** Normalized results.

Date	A	CP	P	W	CB	C	D	HMC	SA	TP	TQ	WL	MA
2010/06	0.452	0.523	0.232	0.256	0.483	0.694	0.545	1.000	0.497	0.651	1.000	0.327	0.429
2010/07	0.741	0.886	0.179	0.442	0.638	0.871	0.610	0.978	0.528	0.860	0.813	0.653	0.971
2010/08	0.645	0.591	0.242	0.093	0.345	0.339	0.260	0.533	0.365	0.349	0.479	0.265	0.771
						…							
2020/10	0.066	0.432	0.242	0.209	0.552	0.613	0.844	0.178	0.541	0.558	0.542	0.469	0.514
2020/11	0.102	0.455	0.126	0.233	0.448	0.371	0.455	0.333	0.333	0.372	0.646	0.449	0.429

**Table 3:** Parameter settings of the ADSGL algorithm.

Parameter	Value
feature dimension	12
output dimension	1
test method	10-fold cross validation
epoch	100
number of groups	2
α	{0.1, 0.3, 0.5, 0.8}

**Table 4:** Comparison of test results with variable selection methods.

Method	RMSE	t/s	Variable_Number
ADSGL	0.058	17.468	3
-	0.116	30.463	12
RR	0.071	24.941	5
LASSO	0.082	30.271	7
Group LASSO	0.072	24.137	6
RF	0.144	27.597	8

## Data Availability

Not applicable.

## References

[1] Liang W.J., Li X.Y. (2018). Flight incidents prediction of air transportation based on the combined model of ARIMA, LS-SVM and BPNN. Saf. Environ. Eng..

[2] Puranik T.G., Rodriguez N., Mavris D.N. (2020). Towards online prediction of safety-critical landing metrics in aviation using supervised machine learning. Transp. Res. Part C Emerg. Technol..

[3] Rma V., Comendador V., Sanz L.P. (2018). Prediction of aircraft safety incidents using Bayesian inference and hierarchical structures-ScienceDirect. Saf. Sci..

[4] Lukáčová A., Babičand F.J., Paralič J. Building the prediction model from the aviation incident data. Proceedings of the IEEE International Symposiumon Applied Machine Intelligence & Informatics.

[5] Zhang X., Mahadevan S. (2019). Ensemble machine learning models for aviation incident risk prediction. Decis. Support Syst..

[6] Qiao X., Chang W., Zhou S., Lu X.F. A prediction model of hard landing based on RBF neural network with K-means clustering algorithm. Proceedings of the 2016 IEEE International Conference on Industrial Engineering and Engineering Management (IEEM).

[7] Xiong M.L., Wang H.W., Xu Y., Fu Q. (2020). General aviation safety research based onprediction of bird strike symptom. Syst. Eng. Electron..

[8] Zhou D., Zhuang X., Zuo H., Wang H., Yan H. (2020). Deep Learning-Based Approach for Civil Aircraft Hazard Identification and Prediction. IEEE Access.

[9] Zhang X.G., Srinivasan P., Mahadevan S. (2021). Sequential deep learning from NTSB reports for aviation safety prognosis. Saf. Sci..

[10] Perboli G., Gajetti M., Fedorov S., Giudice S.L. (2021). Natural language processing for the identification of human factors in aviation accidents causes: An application to the shel methodology. Expert Syst. Appl..

[11] Demirci S. (2022). The requirements for automation systems based on Boeing 737 MAX crashes. Aircr. Eng. Aerosp. Technol..

[12] Hulme A., Stanton N.A., Walker G.H., Waterson P., Salmon P.M. (2019). What do applications of systems thinking accident analysis methods tell us about accident causation? a systematic review of applications between 1990 and 2018. Saf. Sci..

[13] Rong M., Luo M., Chen Y., Sun C., Wang Y. Human Factor Quantitative Analysis Based on OHFAM and Bayesian Network. Proceedings of the International Conference on Human-Computer Interaction, HCI International 2014.

[14] Fu G., Lu B., Chen X.Z. (2005). Behavior Based Model for Organizational Safety Management. China Saf. Sci. J..

[15] O’Connor P., Cowan S., Alton J. (2010). A comparison of leading and lagging indicators of safety in naval aviation. Aviat. Space Environ. Med..

[16] Cui Q., Li Y. (2015). The change trend and influencing factors of civil aviation safety efficiency: The case of Chinese airline companies. Saf. Sci..

[17] Wang S.Z., Ji B.X., Zhao J.S., Liu W., Xu T. (2018). Predicting ship fuel consumption based on LASSO regression. Transp. Res. Part D: Transp. Environ..

[18] Xie J., Hong T. (2018). Variable Selection Methods for Probabilistic Load Forecasting: Empirical Evidence from Seven States of the United States. IEEE Trans. Smart Grid.

[19] Zhao J.C., Deng F., Cai Y.Y. (2019). Long short-term memory-fully connected (LSTM-FC) neural network for pm 2.5 concentration prediction. Chemosphere.

[20] Karmitsa N., Taheri S., Bagirov A., Mäkinen P. (2022). Missing Value Imputation via Clusterwise Linear Regression. IEEE Trans. Knowl. Data Eng..

[21] Xu Y.J., Luo Y.X. (2021). VARCLUS-based Principal Component Lasso Dimensionality Reduction Algorithm and Simulation. Stat. Decis..

[22] Cui H.Y., Xu S., Zhang L.F., Ewelsch R., Kphorn B. (2018). The Key Techniques and Future Vision of variable selection in Machine Learning. J. Beijing Univ. Posts Telecommun..

[23] Daubechies I., Defrise M., Mol C.D. (2004). An iterative thresholding algorithm for linear inverse problems with a sparsity constraint. Commun. Pure Appl. Math..

[24] Fan J., Xue L., Zou H. (2014). Strong oracle optimality of folded concave penalized estimation. Ann. Stat..

[25] Liu H., Mi X., Li Y. (2018). Smart multistep deep learning model for wind speed forecasting based on variational mode decomposition, singular spectrum analysis, LSTM network and ELM. Energy Convers. Manag..

[26] Jiang P., Liu J., Wu F., Wang J., Xue A. (2016). Node Deployment Algorithm for Underwater Sensor Networks Based on Connected Dominating Set. Sensors.

[27] Rose R.L., Puranik T.G., Mavris D.N. (2020). Natural Language Processing Based Method for Clustering and Analysis of Aviation Safety Narratives. Aerospace.

[28] Kabir W., Ahmad M.O., Swamy M.N.S. (2018). Normalization and Weighting Techniques Based on Genuine-Impostor Score Fusion in Multi-Biometric Systems. IEEE Trans. Inf. Forensics Secur..

[29] Fadi T., Suhel H., Firuz K., Amanda G. (2020). Data imbalance in classification: Experimental evaluation. Inf. Sci..

