# Correction: Zeng et al. Research on Aviation Safety Prediction Based on Variable Selection and LSTM. *Sensors* 2023, *23*, 41

**DOI:** 10.3390/s24206605

**Published:** 2024-10-14

**Authors:** Hang Zeng, Jiansheng Guo, Hongmei Zhang, Bo Ren, Jiangnan Wu

**Affiliations:** 1 Equipment Management & UAV Engineering College, Air Force Engineering University, Xi’an 710051, China; 2 Science and Technology on Electro-Optic Control Laboratory, Luoyang 314000, China

## Error in Figure

In the original publication [1]. In the vertical coordinate values of Figure 3, the order should be “−4, −2, 0, 2, 4”; the correct version of Figure 3 appears below:

## Text Correction

There were two errors in the original publication [1].

(1) In the paragraph above Equation (32), “ML-RNN” should be replaced by “MSSRNN”, and “SVM” should be replaced by “SVR (support vector regression)”. The correct paragraph appears below:

To intuitively evaluate the accuracy of the prediction model, single layer-multistep LSTM (LSTM’), the original LSTM, MSSRNN, TCN (temporal convolutional network), DT (decision tree), SVR (support vector regression), and ARMA (autoregressive moving average) predictors are used as control models to compute in the same experimental environment. RMSE is used as the accuracy evaluation indicator. RMSE can be implemented as

(2) In Section 3.2.2, Paragraph 4, the second “Figure 6” should be replaced by “Figure 7”. The correct paragraph appears below:

From Figure 6, it can be seen that: One group of variables’ coefficients (W, CB, C, D, HMC, SA, TP, TQ, WL) converged at a higher rate compared with the other group (A, CP, P), which indicates that the variables in the latter group are still highly interpretable to the output variables at optimal penalty weight. The absolute values of coefficients, which in the first group converged, are depicted in Figure 7.

The authors state that the scientific conclusions are unaffected. This correction was approved by the Academic Editor. The original publication has also been updated.

## Figures and Tables

**Figure 3:**
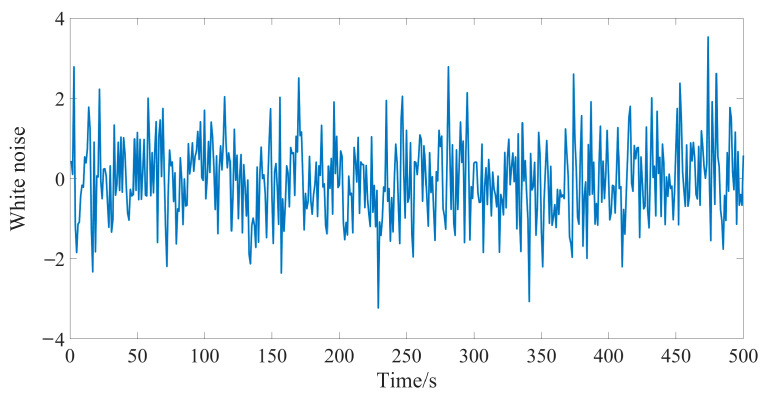
Sequence diagram for white noise.
